# Endoscopic ultrasound avoids adverse events in high probability choledocholithiasis patients with a negative computed tomography

**DOI:** 10.1186/s12876-022-02162-8

**Published:** 2022-03-03

**Authors:** Meng-Ying Lin, Chun-Te Lee, Ming-Tsung Hsieh, Ming-Ching Ou, Yao-Shen Wang, Meng-Chieh Lee, Wei-Lun Chang, Bor-Shyang Sheu

**Affiliations:** 1 Department of Internal Medicine, National Cheng Kung University Hospital, Tainan, Taiwan, R.O.C.; 2 Department of Medical Image, National Cheng Kung University Hospital, Tainan, Taiwan, R.O.C.; 3 Department of Emergent Medicine, National Cheng Kung University Hospital, Tainan, Taiwan, R.O.C.

**Keywords:** Choledocholithiasis, Endoscopic ultrasound, Computed tomography, Endoscopic retrograde cholangiopancreatography

## Abstract

**Background:**

The current guideline recommends patients who meet high probability criteria for choledocholithiasis to receive endoscopic retrograde cholangiopancreatography (ERCP). However, adverse events can occur during ERCP. Our goal is to determine whether endoscopic ultrasound (EUS) before ERCP can avoid unnecessary ERCP complications, especially in patients with a negative CT scan.

**Methods:**

A total of 604 patients with high probability of choledocholithiasis were screened and 104 patients were prospectively enrolled. Patients with malignant biliary obstruction, altered GI anatomy, and choledocholithiasis on CT scan were excluded. Among them, 44 patients received EUS first, and ERCP if choledocholithiasis present (EUS-first group). The other 60 patients received ERCP directly (ERCP-first group). The baseline characteristics, presence of choledocholithiasis, and complications were compared between groups. All patients were followed for 3 months to determine the difference in recurrent biliary event rate. Cost-effectiveness was compared between the two strategies.

**Results:**

There was no marked difference in age, sex, laboratory data, presenting with pancreatitis, and risk factors for choledocholithiasis. Overall, 51 patients (49.0%) had choledocholithiasis, which did not justify the risk of direct ERCP. In the EUS-first group, 27 (61.4%) ERCP procedures were prevented. The overall complication rate was significantly lower in the EUS-first group compared to the ERCP-fist group (6.8% vs. 21.7%, *P* = 0.04). The number-needed-to-treat to avoid one unnecessary adverse event was 6.71. After a 3-month follow-up, the cumulative recurrence biliary event rates were similar (13.6% vs. 15.0%, *P* = 0.803). EUS-first strategy was more cost-effective than the ERCP-first strategy (mean cost 2322.89$ vs. 3175.63$, P = 0.002).

**Conclusions:**

In high-probability choledocholithiasis patients with a negative CT, the EUS-first strategy is cost-effective, which can prevent unnecessary ERCP procedures and their complications.

**Supplementary Information:**

The online version contains supplementary material available at 10.1186/s12876-022-02162-8.

## Background

Most patients with cholelithiasis suffer from no discomfort and is frequently an incidental finding. Among patients who develop symptomatic gallstone disease, only 10–20% has concurent choledocholithiasis [1]. The American Society for Gastrointestinal Endoscopy (ASGE) had established guidelines for classification and managing patients based on risk stratifications in 2010 [2]. Patients who meet either one of the following condition: total bilirubin > 4 mg/dL, total bilirubin 1.8 mg/dL to 4 mg/dL with > 6 mm common bile duct diameter, ascending cholangitis presented clinically, and choledocholithiasis on the transabdominal ultrasound are categorized into the high-probability group. This group is recommended to receive endoscopic retrograde cholangiopancreatography (ERCP) directly. ERCP is effective for removing choledocholithiasis but is highly invasive with 6.9–12% reported adverse event rate [3, 4]. Some of these are life-threatening, such as severe pancreatitis, bleeding, and bowel perforation. Several studies focused on the predictive value of the criteria had shown a positive predictive value ranged from 56.3 to 64% in patients with a high probability of choledocholithiasis [5–7]. It indicates that patients may receive unnecessary ERCP and result in adverse events.

Abdominal computed tomography (CT) scan is often the first diagnostic tool in managing patients suspicious of choledocholithiasis. It is becoming more frequently used due to benefits of being fast, convenient, and non-operator dependent. CT scan can also help to differentiate other etiologies of acute abdomen. However, choledocholithiasis can be small or radiolucent which resulted in an unsatisfied diagnostic sensitivity of CT scan (77.3–85%) [8, 9].

Endoscopic ultrasonography (EUS) and magnetic resonance cholangiopancreatography (MRCP) are excellent tools with a higher sensitivity (93–95%) for diagnosing choledocholithiasis and are less invasive in comparison with ERCP [10]. They had been widely used in patients with an intermediate probability of choledocholithiasis. Endoscopists will find EUS to be more sensitive than MRCP in detecting choledocholithiasis and can be performed prior to ERCP [11, 12]. However, whether EUS is beneficial to high-probability patients has yet to be widely studied. We aimed to conduct a prospective cohort study to analyze if EUS can avoid unnecessary ERCP and its adverse events in patients with a high probability for choledocholithiasis but with a negative CT scan.

## Methods

### Patient enrollment and study design

From January 2018 to August 2019, we prospectively enrolled patients with gallbladder stones who were clinically suspicious of biliary obstruction and met 2010 ASGE high probability criteria for choledocholithiasis. All patients received treatment in a tertiary center in Southern Taiwan with a high annual volume of ERCP (more than 1000 procedures) and EUS (more than 500 procedures). Abdominal CT scan was performed in all enrolled patients. We excluded patients with the following conditions: malignancy-related bile duct obstruction, altered upper gastrointestinal tract anatomy, choledocholithiasis presented on CT scan, unstable vital signs, and ERCP failure. This study was approved by the institutional review board of NCKUH (B-BR-106-066). Written Informed consent was obtained from all participants before initial procedure by principle investigator after fully explaining study protocol to them.

### Patient allocation and data recording

After enrollment, patients’ initial presentation and symptoms, initial laboratory data, and radiological examination results were recorded. Then they were treated according to the preference of the doctor in charge. They could either arrange ERCP directly as suggested by the guideline, or EUS-first to confirm the presence of choledocholithiasis. Initial ERCP and EUS were done within 72 h after enrollment. In patients who received EUS first, therapeutic ERCP will be done within 24 h if EUS reported positive for choledocholithiasis. Those without evidence of stones will receive medical treatment only. The presence of choledocholithiasis was determined by ERCP or EUS (If the patient did not receive ERCP). Patients were admitted to ward and monitored after the procedures. The presence of choledocholithiasis and procedure-associated adverse events were triply confirmed by another two experienced gastroenterologists in the same hospital. After discharge, we routinely followed patients at outpatients department visits or by telephone interviews for 3 months to see if there is any recurrent episodes. Recurrent biliary events including biliary colic, cholecystitis, cholangitis, and biliary pancreatitis were regarded as recurrence in this study. The total inpatient claim including medical examination fee, pharmacy expenses, nursing care fee and general ward fee during hospital stay was recorded and went into cost analysis. Optional self paid part was excluded.

### ERCP and EUS procedure and the definition of adeverse events

All ERCP procedures were done by five certified endoscopists and procedures performed include: cannulation, cholangiography, sphincterotomy or balloon dilation, and stone extraction. EUS procedures were done by two experienced endosonographers using UCT-260 or UE-260-AL5 echoendoscope adapted to Olympus EUS ME-1 or ME-2 premium system. The presence of choledocholithiasis on EUS was defined as reproducible hyperechoic materials with acoustic shadow in the extrahepatic duct.

The definition of post ERCP pancreatitis used in this study was “new or worsened abdominal pain combined with > 3 times the normal value of amylase or lipase at more than 24 h after ERCP and requirement of admission or prolongation of a planned admission” [13, 14]. Immediate bleeding during ERCP procedure requiring endoscopic treatment and clinically relevant hematemesis and/or melena in any degree up to 14 days after procedure were defined as post ERCP bleeding [15, 16]. Post ERCP ascending cholangitis was defined as “new onset temperature > 38 °C for more than 24 h combined with cholestasis” [17]. Other less frequent adeverse events were defined according to previous guideline [17].

### CT image interpretation

Choledocholithiasis varies in density on CT. The unenhanced CT images were interpreted first for detecting radiopaque materials in the common bile duct. Then, the contrast-enhancing CT series were viewed to detect biliary wall thickening which suggested cholangitis and required a second-look for radiolucent materials inside. By using this principle, the presence of choledocholithiasis on the CT scan was primarily reported by on duty radiologist and confirmed later by another experienced radiologist who specializes in abdominal image.

### Presentation of choledocholithiasis

We defined the presentation of choledocholithiasis by ERCP result. Only patients with obvious stone or sludge that was recorded on ERCP images and confirmed by second doctor were defined as CBD stone positive cases. Patients with a negative ERCP result or revealed no stone by EUS exam were defined as CBD stone negative cases.

### Statistical analysis

All statistical analyses were performed on the SPSS software (version 20.0, IBM Corporation, Armonk, NY, USA). The difference in initial presentations and symptoms, initial laboratory data, and enrollment criteria was analyzed by Student t-test or χ^2^ test accordingly. The difference in adverse event and choledocholithiasis rates between groups were analyzed by the χ^2^ test. The disease recurrence rate for each group was estimated by the Kaplan–Meier curve and compared by log-rank test.

## Results

### The baseline characteristics and laboratory data of the enrolled patients

**Fig. 1:**
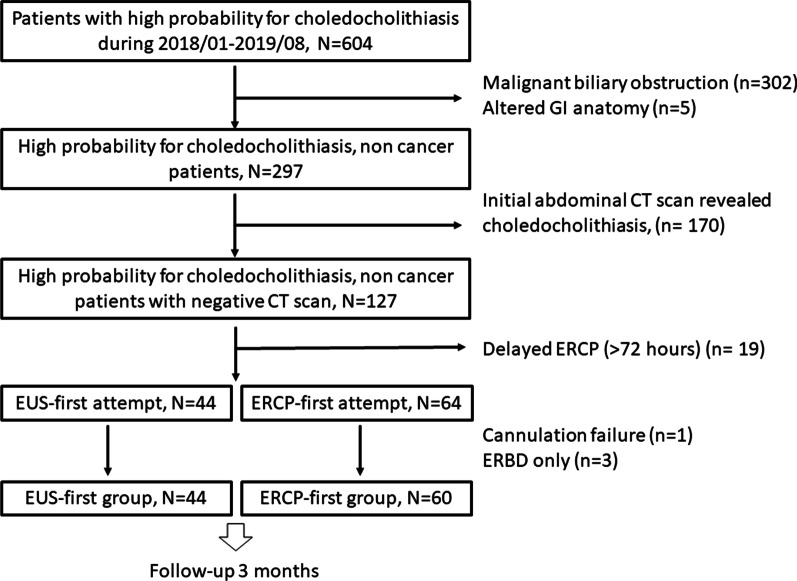
The flow chart of patient enrollment. The exclusion criteria and case numbers excluded were listed on the side

A total of 104 patients with gall bladder stones and met criterias were included (44 received EUS-first and 60 received ERCP-first). The detailed enrollment algorithm was illustrated in Fig. 1. The baseline characteristics were listed in Table 1. There was no significant difference between groups including age, sex, laboratory data such as AST, ALT, total bilirubin, alkaline phosphatase, and the presence of pancreatitis. However, the CBD diameter and treated papilla ratio were higher in ERCP first group.

**Table 1:** Baseline characteristics of the enrolled patients

	EUS-first (n = 44)	ERCP-first (n = 60)	*P* value
Age (years) mean ± SD	59.04 ± 15.82	62.82 ± 19.89	0.30
Sex (male/female)	24/20	37/23	0.47
AST (U/L) mean ± SD	334.48 ± 274.56	354.47 ± 219.62	0.68
ALT (U/L) mean ± SD	347.59 ± 312.21	397.57 ± 227.73	0.38
Total bilirubin (mg/dL) mean ± SD	3.74 ± 2.34	3.62 ± 1.79	0.77
ALK-P (U/L) mean ± SD	207.55 ± 169.02	215.35 ± 90.18	0.80
Pancreatitis (yes/no)	(14/30)	(17/43)	0.70
CBD diameter (mm) (median, IQR)	7.0 (4.25–8.00)	8.5 (7.0–11.0)	< 0.001
Naïve papilla (Yes/No)	44/0	53/7	0.02
Diffcullt cannulation (%)			0.787
Main P duct cannulation	2 (11.8)	12 (20)	
Cannulation > 10 min	1 (5.9)	4 (6.7)	
> 5 times trial	0	1 (1.7)	
P duct injection	0	2 (3.3)	
P duct stent (%)	1 (5.9)	13 (21.7)	0.173
Sphincterotomy method			< 0.001
EST*	15	34	
EPBD^†^	2	16	
Pre-cut or TPS^‡^	0	10	
EUS only	27	0	

*EST, endoscopic sphincterotomy

^†^EPBD, endoscopic papillary balloon dilation

^‡^TPS, trans-pancreatic sphincterotomy

### The distribution of ASGE criteria and common risk factors for choledocholithiasis

The kinds of ASGE high probability criteria met in our patients were recorded in Table 2. There was no significant difference in the presence of each criterion between groups. Table 2 also shows the common risk factors (forty, fat, fertility, and female) for choledocholithiasis of the enrolled patients. Again, there was no significant difference between groups.

**Table 2:** The risk factors and high probability criteria met in the two groups

	EUS-first (n = 44)	ERCP-first (n = 60)	P value
**Risk factor**			
4F* scale (0–4)	4/18/7/14/1	3/35/8/11/3	0.32
Forty (%)	35 (79.5)	45 (75.0)	0.59
Fertility (%)	17 (38.6)	19 (31.7)	0.46
Fatty (%)	6 (13.6)	9 (15.0)	0.85
Female (%)	20 (45.5)	23 (38.3)	0.47
**The high probability criteria met**			
Total bilirubin > 4 (mg/dL)	11 (25.0)	19 (31.7)	0.46
Clinical ascending cholangitis	9 (20.5)	11 (18.3)	0.79
Choledocholithiasis on ultrasound	1 (2.3)	1 (1.7)	0.82
Total bilirubin 1.8–4 (mg/dL) & CBD^†^ > 6 mm	28 (63.6)	36 (60.0)	0.71

*4F, forty, fertility, fatty, and female

^†^CBD, common bile duct

### The presence of choledocholithiasis and procedure-related adverse events

Overall, 51 patients (49.0%) had choledocholithiasis, including 13 patients (29.5%) in the EUS-first group and 38 patients (63.3%) in the ERCP-fist group. In the EUS-first group, 17 patients underwent ERCP procedure, and 27 of 44 (61.4%) ERCP procedures were successfully avoided. In our cohort, EUS detected all choledocholithiasis, with only 4 false-positive cases proved by ERCP later. In this study, the EUS-sensitivity and specificity for choledocholithiasis diagnosis was 100% and 87.1%, respectively. In Table 3, the overall adverse event rate was lower in the EUS-first group (6.8% versus 21.7%, *P* = 0.04). Bleeding and ascending cholangitis were the most common adverse events in this study. The events presented in the EUS-first group all resulted from the subsequent ERCP procedures. The numbers-needed-to-treat to avoid one adverse event was 6.71, which means in the high-probability choledocholithiasis patients with a negative CT scan, every 7 EUS procedures can avoid one adverse event. The detailed incidence of other adverse events were listed in Table 3.

**Table 3:** The detailed adverse events and recurrent episodes in the two groups

	EUS-first (n = 44)	ERCP-first (n = 60)	P value
All events (%)	3 (6.8)	13 (21.7)	0.04
Pancreatitis			
Mild to moderate	1 (2.3)	1 (1.7)	
Severe	0	0	
Bleeding			
Endoscopic treatment	1 (2.3)	8 (13.3)	
TAE* or surgical treatment	0	0	
Ascending cholangitis			
Fever or sepsis	0	3 (5.0)	
Septic shock	1 (2.3)	0	
Others	0	1^†^ (1.7)	
NNT^‡^ for avoiding complications	6.71		

	Recurrent episode within 3 months			
	EUS (n = 27)	EUS + ERCP (n = 17)	ERCP (n = 60)	*P value*
Total recurrence	2	4	9	0.12
Biliary colic	1	2	1	
Cholecystitis	1	0	6	
Recurrent CBD stone	0	2	2	
Biliary pancreatitis	0	0	0	

*TAE, trans-arterial embolization

^†^Duodenal perforation

^‡^NNT, number needed to treat

### The recurrent biliary events within 3 months

**Fig. 2:**
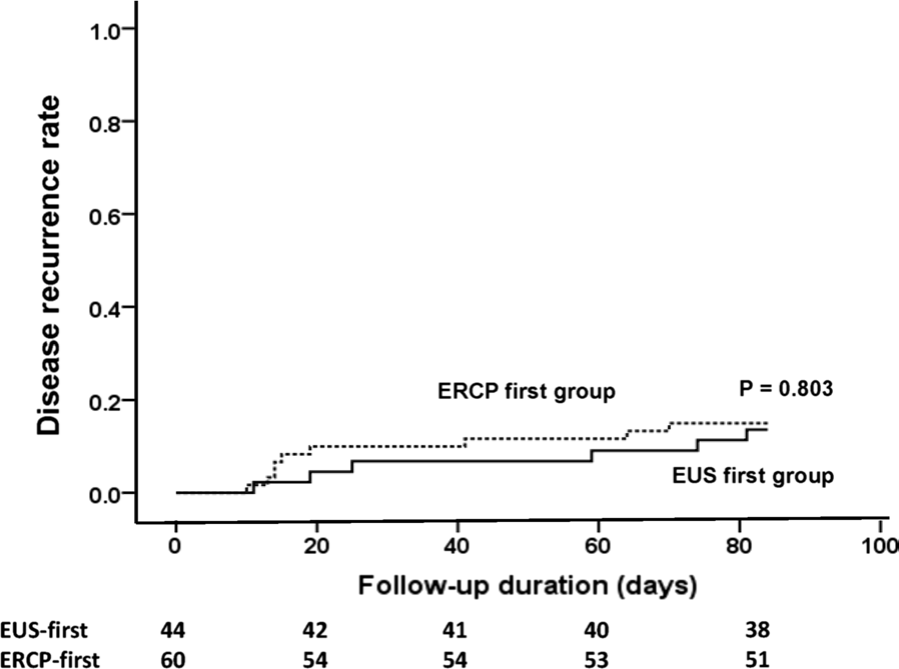
The disease recurrence rate within 3 months of follow-up. The dotted line represents the ERCP-first group, and the solid line stands for the EUS-first group. No obvious difference in the recurrence rate was observed between the two groups

During a 3-month follow-up, a total of 15 recurrence episodes (6 in the EUS-first group and 9 in the ERCP-first group) were recorded. Among them, 4 episodes (2 in the EUS-first group and 2 in the ERCP-first group) were associated with recurrent choledocholithiasis, 7 patients had acute cholecystitis, and 4 patients had biliary colic without evidence of choledocholithiasis. The detail diagnosis of recurrent cases were recorded in Table 3. The 3-month cumulative recurrence rate was not significantly different between groups as illustrated in Fig. 2 (*P* = 0.803).

### The inpatient days and cost effectiveness analysis

As illustrate in Table 4, patients in the EUS-first group had similar inpatient days compared with ERCP-first group (6.00 vs. 6.78, p = 0.27). However, the medical cost (in US dollar) was much lower in the EUS-first group (mean cost 2322.89$ vs. 3175.63$, P = 0.002). The medical cost was even lower in patients who receive EUS exam only in this clinical scenario (1560.74$). As a result, EUS-first strategy will still be cost-effectiveness in a population whose choledocholithiasis rate is equal to or lower than 71.9%.

**Table 4:** The length of hospital stay and medical cost

	EUS-first (n = 44)	ERCP-first (n = 60)	P value
Inpatient days	6.00 ± 3.62	6.78 ± 3.52	0.27
(Mean ± SD)			
Medical cost*			
(Mean ± SD)			
Overall	2322.89 ± 1517.88	3175.63 ± 1194.18	0.02
EUS only (n = 27)	1560.74 ± 992.40		
EUS + ERCP (n = 17)	3533.35 ± 1444.49		

*In US dollars

## Discussion

Choledocholithiasis is a common and emergent disease worldwide. Patients who have choledocholithiasis need invasive procedures such as therapeutic ERCP or surgery to treat [18]. Several criteria based on biochemical tests and transabdominal ultrasound had been proposed to select patients with a high probability of choledocholithiasis [7, 19–22], but the accuracy was unsatisfactory, ranging from 38.7 to 64%. As a result, invasive ERCP is unnecessary in some of these patients, which can lead to unwanted adverse events.

To the best of our knowledge, we are the first to clarify the role of EUS in patients with a high probability of choledocholithiasis and a negative CT scan. In these patients, EUS before therapeutic ERCP can prevent unnecessary ERCP and preventable adverse events. We found the positive rate of choledocholithiasis was less than 50% (only 49.0% in our cohort). According to the current ASGE guideline, in patients with a probability of 10–50% for choledocholithiasis, a confirmative test (such as EUS or MRCP) is suggested before ERCP. Similar to our findings, one previous study also showed EUS-first strategy can avoid 57.5% ERCP in patients with a high probability of choledocholithiasis [23]. Our study further demonstrated that the EUS-first strategy can significantly decrease adverse event rate from 21.7 to 6.8% (*P* = 0.04). EUS-first policy in these patients not only reduced adverse events, but was also cost-effectiveness, especillay in countries where the EUS cost much less than ERCP. As demonstrated in our cohort, patients who received EUS plus ERCP had slightly higher cost than ERCP-first group (3533.35$ vs. 3175.63$). However, the cost reduction in the EUS-only patients was tremendous (1560.74$ vs. 3175.63$). Taken these factors into consideration, EUS-first strategy will still be cost-effectiveness in patients with a choledocholithiasis-presenting rate as high as 71.9%.

We found a lower rate of choledocholithiasis (49.0%) in the high-probability patients compared to previous reports (56.3–64%) [5–7]. Take note that if we enrolled all high-probability patients, up to 74.4% (221/297) of them may have choledocholithiasis. Since patients with radiopaque choledocholithiasis was successfully detected by CT scan and excluded from this study. Less than half of the remaining high-probability patients had choledocholithiasis; the end does not justify the risk taken for unnecessary ERCP.

Our study showed the ERCP-first group had a higher rate of choledocholithiasis than the EUS-first group (63.3% vs. 29.5%, *P* = 0.001). This may result from the non-randomized study design and the fact that the in-charge doctor chose a therapeutic strategy based on the clinical condition. As illustrated in Table 1, Patients in EUS-first group had smaller caliber of CBD which had been reported to associate with choledocholithiasis presentation [24]. In addition, as revealed in Additional file 1: Table S1, more patients in the EUS-first group (68.2% vs. 28.3%, *P* < 0.001) received a second set of biochemistry exams before subsequent endoscopic procedures, and data improvement was noted in a high percentage (82.1–89.7%) of the EUS-fist group. It had been shown that when patients displaying a laboratroy improvement to the level as those with intermediate- or low-probability, only 34.1% had choledocholithiasis [7]. Therefore, clinical improvement may indicate a passed gallstone and influenced the decision making of the in-charge doctor in our study. Despite a higher choledocholithiasis rate in the ERCP-first group compared to the EUS-first group, we showed a comparable 3-month disease recurrence rate in both groups.

The cumulative rate of recurrent biliary events within 3 months seemed high in both groups (13.6% and 15% separately). However, if we restrict recurrent episodes to CBD stone recurrence, the recurrent rate decreased to 4.5% and 3.3%, respectively, which was lower than previous reports [25]. We also found that all the CBD stone recurrent episodes happened in the post-ERCP patients. Since all ERCP procedures were conducted by highly-experienced endoscopists who routinely confirmed completion of stone removal by repeating cholangiogram, we supposed that the recurrent episodes was caused by recurrent cholelithiasis rather than residual stone. To exlude the possibility of false negative in the EUS-only group, we have followed patients for 3 months. As a result, there were no CBD stone recurrence in the EUS-only group. The most common recurrent episodes in the EUS-only group were biliary colic, suggesting that even if there is tiny missed CBD stones on EUS, they would only cause minor symptoms then passed through spontaneously. Taken together, these data suggested that the EUS-first strategy is safer and equally effective in treating the high-probability choledocholithiasis patients with a negative CT scan.

The bleeding rate in the ERCP-first group (13.3%) is higher compared to previous studies (0.3–9.6%) [17]. The definition for post-ERCP bleeding was not universal which made the reported incidence of post-ERCP bleeding varied [16, 26]. Second, the higher sphincterotomy rate (79.2%), a validated risk factor for post-ERCP bleeding in our study may also lead to the higher post-ERCP bleeding rate [27, 28]. Third, our study population had more naïve papilla (70/77, 90.1%) and difficult cannulation cases (18/77, 23.4%), which resulted in a higher transpancreatic sphincterotomy/needle knife sphincterotomy rate (10/77, 13.0%). These may also contribute to the higher bleeding adverse events [29, 30].

Several limitations are noted in our study. First, the observational study design resulted in uneven choledocholithiasis rate in the two groups. Therefore, further randomized clinical trial is warranted to validate our findings. Second, the case number is small due to the strict criteria used for patient enrollment. We focused on high-probability patients with a negative CT scan, thus 83% of patients were excluded from the study. Despite a small case number, we had successfully demonstrated a significantly lower adverse event rate in the EUS-first group.

## Conclusion

In conclusion, we recommend routine use of EUS before therapeutic ERCP in patients with a high probability of choledocholithiasis but negative CT scan, especially when clinically improving. This strategy can reduce unnecessary ERCP procedures and their adverse events. EUS prior to ERCP is also cost–benefit in area where EUS cost much less than ERCP.

## Supplementary Information

**Additional file 1: Table S1.** The presented 2nd set of laboratory data before any procedure and its trend.

## Data Availability

The datasets used and analysed during the current study are available from the corresponding author on reasonable request.

## Abbreviations

CT: Computed tomography
ERCP: Endoscopic retrograde cholangiopancreatography
EUS: Endoscopic ultrasound
GI: Gastrointestinal
ASGE: American Society for Gastrointestinal Endoscopy
NCKUH: National Cheng Kung University Hospital
MRCP: Magnetic resonance cholangiopancreatography
AST: Aspartate Transaminase
ALT: Alanine aminotransferase
