# Supplementary material for: Endoscopic ultrasound avoids adverse events in high probability choledocholithiasis patients with a negative computed tomography
Source: BMC Gastroenterol. 2022 Mar 3;22:94. doi: 10.1186/s12876-022-02162-8 (PMC8895914; doi:10.1186/s12876-022-02162-8)
Supplement: Supplementary file 1 — Additional file 1: Table S1. The presented 2nd set of laboratory data before any procedure and its trend. [file 12876_2022_2162_MOESM1_ESM.docx]

| **Supplement Table 1: The presented 2^nd^ set of laboratory data before any procedure** | | | |
| --- | --- | --- | --- |
|  | EUS-first | ERCP-first | *P* value |
| Presented with 2^nd^ set of lab data before procedure (%) | 30/44 (68.2) | 17/60 (28.3) | < 0.001 |
| AST^*^ decreased case (%) | 26/29 (89.7) | 12/15 (80) | 0.39 |
| ALT^†^ decreased case (%) | 23/28 (82.1) | 10/15 (66.7) | 0.25 |
| Total bilirubin decreased case (%) | 26/29 (89.7) | 11/16 (66.7) | 0.11 |
